# Fenton Reaction-Induced Oxidative Damage to Membrane Lipids and Protective Effects of 17β-Estradiol in Porcine Ovary and Thyroid Homogenates

**DOI:** 10.3390/ijerph17186841

**Published:** 2020-09-18

**Authors:** Aleksandra Rynkowska, Jan Stępniak, Małgorzata Karbownik-Lewińska

**Affiliations:** 1 Department of Oncological Endocrinology, Medical University of Łódź, 90-752 Łódź, Poland; aleksandra.rynkowska@umed.lodz.pl (A.R.); jan.stepniak@umed.lodz.pl (J.S.); 2 Polish Mother’s Memorial Hospital—Research Institute, 93-338 Łódź, Poland

**Keywords:** 17β-estradiol, Fenton reaction, ferrous, lipid peroxidation, oxidative damage

## Abstract

The Fenton reaction (Fe^2+^+H_2_O_2_→Fe^3+^+^•^OH+OH^-^) results in strong oxidative damage to macromolecules when iron (Fe) or hydrogen peroxide (H_2_O_2_) are in excess. This study aims at comparing Fe^2+^+H_2_O_2_-induced oxidative damage to membrane lipids (lipid peroxidation, LPO) and protective effects of 17β-estradiol (a potential antioxidant) in porcine ovary and thyroid homogenates. Iron, as one of the Fenton reaction substrates, was used in the highest achievable concentrations. Thyroid or ovary homogenates were incubated in the presence of: (1st) FeSO_4_+H_2_O_2_ with/without 17β-estradiol (1 mM; 100, 10.0, 1.0 µM; 100, 10.0, 1.0 nM; 100, 10.0, 1.0 pM); five experiments were performed with different FeSO_4_ concentrations (2400, 1200, 600, 300, 150 µM); (2nd) FeSO_4_ (2400, 1200, 600, 300, 150 µM)+H_2_O_2_ with/without 17β-estradiol; three experiments were performed with three highest 17β-estradiol concentrations; (3rd) FeSO_4_ (2400, 1200, 1100, 1000, 900, 800, 700, 600, 300, 150, 75 µM)+H_2_O_2_ (5 mM). LPO level [MDA+4-HDA/mg protein] was measured spectrophotometrically. The basal LPO level is lower in ovary than in thyroid homogenates. However, experimentally-induced LPO was higher in the former tissue, which was confirmed for the three highest Fe^2+^ concentrations (2400, 1200, 1100 µM). Exogenous 17β-estradiol (1 mM, 100, and 10 µM) reduced experimentally-induced LPO independently of iron concentration and that protective effect did not differ between tissues. The ovary, compared to the thyroid, reveals higher sensitivity to prooxidative effects of iron, however, it showed similar responsivity to protective 17β-estradiol activity. The therapeutic effect of 17β-estradiol against iron overload consequences should be considered with relation to both tissues.

## 1. Introduction

Despite the fact that reactive oxygen species (ROS) are necessary for proper functioning of organisms, some of them are very harmful, with the hydroxyl radical (^•^OH) being most dangerous. The ^•^OH reacts—at a diffusion-controlled rate—with almost all biological molecules in living cells, forming products which contribute to further damage of biological structures [1]. It is the main product of the so-called Fenton reaction when hydrogen peroxide (H_2_O_2_) interacts with cations of a transition metal, such as iron (Fe^2+^+H_2_O_2_→Fe^3+^+^•^OH+OH^-^) [2]. It is worth mentioning that the original experiment was performed by Fenton more than 120 years ago. In this experiment tartaric acid in aqueous solution interacted with certain oxidizing agents in presence of a ferrous salt, producing a solution “which gives a beautiful violet color on the addition of caustic alkali” [3]. Interestingly, the author did not know at that time that this reaction yielded the ^•^OH. Concerning the clinical relevance of the damaging effects of the ^•^OH, it has been confirmed with relation to such pathological conditions as cancer, atherosclerosis, or neurodegenerative diseases [4].

Playing a role of an important micronutrient in the human body, iron is a cofactor for many biological reactions. Therefore, its presence is also essential for the proper functioning of all organs, the thyroid gland and the ovaries included. However, an excess of iron can contribute to increased oxidative stress leading to numerous pathological conditions. For example, in thyroid follicular cells, excess of iron can disturb hormonogenesis [5]. Concerning the ovaries, iron excess can directly suppress female gonadal function [6]. Both substrates of the Fenton reaction (Fe^2+^ and H_2_O_2_) can be used to experimentally induce oxidative damage in macromolecules, such as lipids, protein, and DNA [7,8,9,10,11,12].

The increased oxidative stress can be reduced by exo- and endogenous antioxidants. Humans obtain some antioxidants from the diet. Additionally, the human body produces many antioxidants themselves, like amino acids or hormones. One of the hormones with antioxidative properties is 17β-estradiol—the main representative of estrogens. The ability of 17β-estradiol to reduce oxidative stress results, among others, from its chemical structure containing four cycloalkane rings with an A-ring responsible for potential antioxidative effects [13]. It has been observed in the above study that 17β-estradiol can prevent intracellular peroxide accumulation and, ultimately, the degeneration of primary neurons, clonal hippocampal cells, and cells in organotypic hippocampal slices [13]; similar effects can be considered in other tissues, such as the thyroid or the ovary. Moreover, 17β-estradiol has an ability to regenerate after scavenging free radicals [14]. Protective antioxidative properties of 17β-estradiol were confirmed, among others, in our experimental studies [10,15].

Theoretically, other potential mechanisms also responsible for antioxidative effects of 17β-estradiol should be considered, such as receptor-mediated effects. Whereas the ovary belongs to organs with the highest level of estrogen receptors, such as ERα and ERβ [16], the thyroid gland possesses the same receptors [17] but probably at lower levels; unfortunately, no comparative studies were performed concerning this issue. It should be stressed, however, that there is no clear evidence in the literature for receptor-dependent antioxidative effects of estrogens. Instead, receptor-independent antioxidative mechanisms of estrogens were documented [18].

The purpose of the study was to compare levels of oxidative damage to membrane lipids (lipid peroxidation, LPO) induced by Fenton reaction substrates (Fe^2+^+H_2_O_2_) and to compare protective antioxidative effects of 17β-estradiol in porcine ovary and thyroid homogenates. The study was performed on raw, uncharacterized tissue homogenates. Iron, as one of the Fenton reaction substrates, was used in the highest achievable concentrations, i.e., ten-fold higher than it was used routinely in published studies. The ovarian and thyroid tissues were chosen because these endocrine glands seem to differ substantially concerning oxidative processes, as suggested by the results of our earlier studies [9,11].

## 2. Materials and Methods 

### 2.1. Chemicals

17β-Estradiol, ferrous sulfate (FeSO_4_), and hydrogen peroxide (H_2_O_2_) were purchased from Sigma (St. Louis, MO, USA). Ethanol (96%) was purchased from Stanlab (Lublin, Poland). The LPO-586 kit for lipid peroxidation was obtained from Enzo Life Science (Farmingdale, NY, USA). All the used chemicals were of analytical grade and came from commercial sources.

### 2.2. Animals

Female porcine thyroids and ovaries were collected from twenty four (24) animals at a slaughter-house, frozen on solid CO_2_, and stored at –80 °C until assay.

### 2.3. Assay of Lipid Peroxidation

Thyroid and ovary tissues were homogenized in ice cold 50 mM Tris-HCl buffer (pH 7.4) (10%, *w*/*v*), and then incubated for 30 min at 37°C in the presence of examined substances. 17β-estradiol was dissolved in ethanol.

In the 1st set of experiments homogenates were incubated in the presence of FeSO_4_ + H_2_O_2_ (5 mM) with or without 17β-estradiol (1 mM; 100, 10.0, 1.0 µM; 100, 10.0, 1.0 nM; 100, 10.0, 1.0 pM). Five experiments were performed with different concentrations of FeSO_4_, i.e., 2400, 1200, 600, 300, and 150 µM. Each experiment was run in duplicate and repeated three times.

In the 2nd set of experiments homogenates were incubated in the presence of FeSO_4_ (2400, 1200, 600, 300, and 150 µM) + H_2_O_2_ (5 mM) with or without 17β-estradiol. Three experiments were performed with these three concentrations of 17β-estradiol, which revealed protective effects in the 1st set of experiments, i.e., 1 mM, 100 µM, and 10 µM. Each experiment was run in duplicate and repeated three times.

The 3rd set of experiments was designed to clarify the differences between tissues concerning Fenton reaction-induced lipid peroxidation, observed in the 1st and in the 2nd sets of experiments. Thus, in the 3rd set of experiments homogenates where incubated in the presence of: FeSO_4_ (2400, 1200, 1100, 1000, 900, 800, 700, 600, 300, 150, and 75 µM) + H_2_O_2_ (5 mM). Each experiment was run in duplicate and repeated five times.

### 2.4. Measurement of Lipid Peroxidation Products

The concentration of malondialdehyde + 4-hydroxyalkenals (MDA+4-HDA), as an index of LPO, was measured in tissue homogenates, as described elsewhere [12]. Protein was measured, using the method of Bradford [19].

In our pilot study, the use of ferrous ions (Fe^2+^) in concentration of 4800 µM resulted in dramatic decrease in spectrophotometric absorption, probably associated with an interference with the method. Therefore, the highest concentration of FeSO_4_ used in our study was 2400 µM.

### 2.5. Statistical Analyses

The data were statistically analyzed, using a one-way analysis at variance (ANOVA), followed by the Student–Neuman–Keuls’ test, or using an unpaired t-test. Statistical significance was determined at the level of *p* < 0.05. Results are presented as means ± SE.

**Figure 1:**
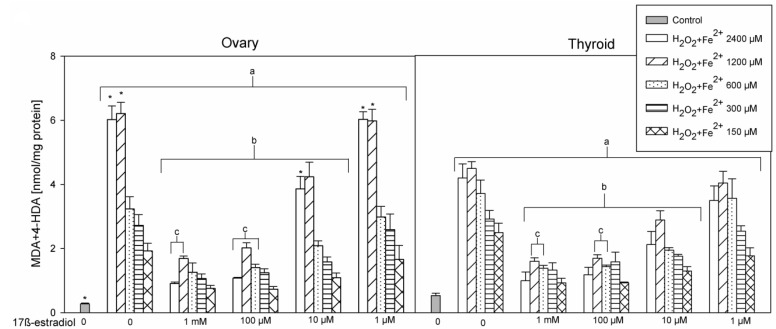
The concentrations of malondialdehyde + 4-hydroxyalkenals (MDA+4-HDA) in porcine ovary and thyroid homogenates. Homogenates were incubated in the presence of FeSO_4_ (2400, 120, 600, 300, and 150 µM) plus H_2_O_2_ (5 mM) used to induce lipid peroxidation and, additionally, in the presence of 17β-estradiol (1 mM; 100, 10.0, 1.0 µM; 100, 10.0, 1.0 nM; 100, 10.0, 1.0 pM). Only four concentrations of 17β-estradiol are presented in Figure 1, as other concentrations revealed exactly the same effects as the concentration of 1 µM. Data are expressed as the amount of MDA+4-HDA (nmol) per mg of protein. Bars represent the mean ± SE of three independent experiments run in duplicates. ^a^ *p* < 0.05 vs. control ‘0’ in the same tissue; ^b^ *p* < 0.05 vs. respective concentration of Fe^2+^ (+H_2_O_2_) without 17β-estradiol; ^c^ *p* < 0.05 vs. respective concentration of Fe^2+^ (+H_2_O_2_) + 17β-estradiol 10 µM; * *p* < 0.05 vs. the same substance/concentration in the other tissue.

**Figure 2:**
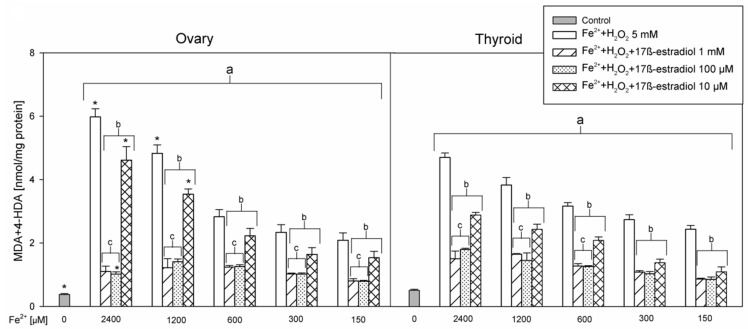
The concentrations of malondialdehyde + 4-hydroxyalkenals (MDA+4-HDA) in ovary and thyroid homogenates. Homogenates were incubated in the presence of FeSO_4_ (2400, 1200, 600, 300, and 150 µM) plus H_2_O_2_ (5 mM) with or without 17β-estradiol (1 mM, 100 µM and 10 µM). Data are expressed as the amount of MDA+4-HDA (nmol) per mg of protein. Bars represent the mean ± SE of three independent experiments run in duplicates. ^a^ *p* < 0.05 vs. control ‘0’ in the same tissue; ^b^ *p* < 0.05 vs. respective concentration of Fe^2+^ (+H_2_O_2_) without 17β-estradiol; ^c^ *p* < 0.05 vs. respective concentration of Fe^2+^ (+H_2_O_2_) + 17β-estradiol 10 µM; * *p* < 0.05 vs. the same substance/concentration in the other tissue.

**Figure 3:**
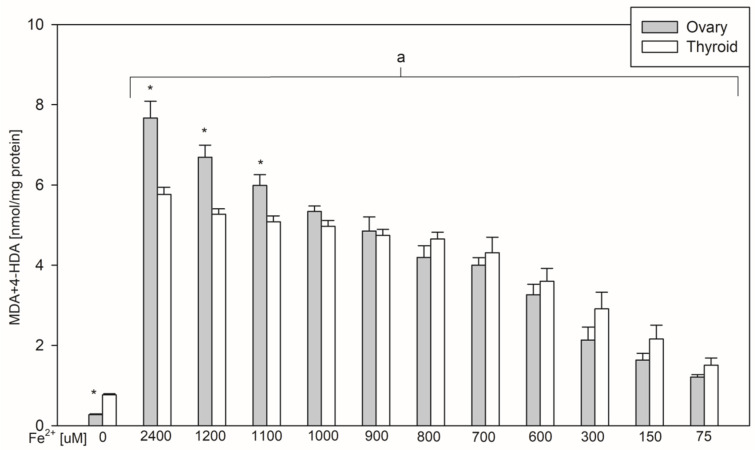
The concentrations of malondialdehyde + 4-hydroxyalkenals (MDA+4-HDA) in porcine ovary and thyroid homogenates. Homogenates were incubated in the presence of FeSO_4_ (2400, 1200, 1100, 1000, 900, 800, 700, 600, 300, 150, and 75 µM) plus H_2_O_2_ (5 mM) used to induce lipid peroxidation. Data are expressed as the amount of MDA+4-HDA (nmol) per mg of protein. Bars represent the mean ± SE of five independent experiments run in duplicates. ^a^ *p* < 0.05 vs. control ‘0’ in the same tissue; * *p* < 0.05 vs. the same concentration in the other tissue.

In the 1st set of experiments results of five experiments with different concentrations of FeSO_4_ were pooled and are presented in Figure 1. In the 2nd set of experiments results of three experiments with different concentrations of 17β-estradiol were pooled and are presented in Figure 2. Results of the 3rd set of experiments, comprising only one experiment with different concentrations of FeSO_4_, are presented in Figure 3.

## 3. Results

The basal level of lipid peroxidation (control ‘0’) was lower in ovary than in thyroid homogenates, which was confirmed in all three sets of experiments (Figure 1, Figure 2 and Figure 3). Oppositely, Fenton reaction-induced lipid peroxidation was higher in ovary than in thyroid homogenates, and that was observed for the three highest concentrations of Fe^2+^, i.e., 2400, 1200, and 1100 µM (Figure 3).

In the 1st set of experiments, 17β-estradiol reduced the level of Fenton reaction-induced oxidative damage in iron concentration-dependent manner. Statistical significance was found for three highest used concentrations of 17β-estradiol, i.e., for 1 mM, 100 µM, and 10 µM, with concentration of 1 mM and 100 µM revealing stronger protective effects than that one of 10 µM (Figure 1). Protective effect of 17β-estradiol did not depend on FeSO_4_ concentration, was effective against all five used FeSO_4_ concentrations and did not differ significantly between thyroid and ovary homogenates. 

In the 2nd set of experiments, three highest concentrations of 17β-estradiol, which revealed protective effects in the 1st set of experiments, were used. Similarly, as it was found in the 1st set of experiments, protective effects of 17β-estradiol was concentration-dependent and stronger for concentrations of 1 mM and 100 µM than for the concentration of 10 µM. Furthermore, protective effects of 17β-estradiol did not depend on FeSO_4_ concentration and did not differ between thyroid and ovary homogenates (Figure 2).

In the 3rd set of experiments it has been observed that Fenton reaction-induced lipid peroxidation is significantly higher in ovary than in thyroid homogenates. This statistically significant difference was confirmed for three highest concentrations of Fe^2+^, i.e., 2400, 1200, and 1100 µM (Figure 3).

## 4. Discussion

Our earlier observations from two different studies [9,11], showing that the basal level of lipid peroxidation is higher in thyroid than in ovary homogenates, was confirmed in the present study. In contrast, lipid peroxidation induced by Fenton reaction substrates was higher in ovary than in thyroid homogenates. This is in accordance with a hypothesis that oxidative stress in the thyroid is at a ‘higher level’ than in other tissues, resulting from oxidative reactions indispensable for thyroid hormone synthesis [8]. This can be also associated with a presumably (although not confirmed in experimental studies) high concentration of iron present in heme-linked histidine residue of thyroid peroxidase [5]. At the same time, this higher concentration of endogenous iron in the thyroid may be responsible for the weaker response to exogenous iron in the thyroid than in the ovaries, observed in the present study. Thus, the important point is that the ovary tissue can face higher oxidative damage in response to exogenous iron or other prooxidative agents. 

In all studies published earlier iron was used in concentrations not exceeding the value of 300 µM [7,8,9,10,11,12] and an iron concentration-dependent effect was observed [9,10,11]. In the present work, FeSO_4_ was used in tenfold higher concentrations (the highest achievable concentrations under in vitro conditions) than in previous studies.

Patients with asymptomatic and symptomatic hemochromatosis are characterized by hepatic iron concentration estimated to be 36–550 μmol/g dry weight tissue (36–550 mM), while the normal hepatic concentration is 5–36 μmol/g (5–36 mM). In turn, blood iron concentration is 150–300 μg/dL (0.026–0.053 mM) in patients with hemochromatosis, while in healthy subjects it is below 150 μg/dL [20]. Neither physiological nor pathological iron concentrations in the thyroid and in the ovary have been estimated precisely till now, however, they are possibly much higher than those in the blood.

Thus, the range of iron concentrations used in the present study (0.15–2.4 mM), exceeding that in the blood, may correspond to iron accumulation in any tissue under conditions of iron overload.

The increased iron stores in the organism are associated with the occurrence of harmful oxidative stress [21]. Therefore, protective antioxidative effects are crucial for proper functioning of the organism. Due to its biochemical properties 17β-estradiol may serve as an effective antioxidant.

In the present study we have found a reduction in lipid peroxidation due to 17β-estradiol treatment. The protective effect of 17β-estradiol was independent of iron concentration and was observed for three highest concentrations of 17β-estradiol, i.e., 1 mM, 100 µM, and 10 µM. However, it should be stressed that these concentrations of 17β-estradiol, which revealed protective effects in our model, can only be achieved under experimental conditions. The highest physiologically achievable concentration of 17β-estradiol is estimated to be approx. 300 pg/mL (1 nM) during midcycle in premenopausal women, and during pregnancy—to even 7000 pg/mL (25 nM) [22].

It is reasonable to consider that the lack of any positive effects of 17β-estradiol used in lower concentrations may result from the solvent used. In our study 17β-estradiol was dissolved in ethanol. It should be stressed that, in other in vitro studies, 17β-estradiol (either dissolved in ethanol or in DMSO or when a solvent was not specified) used in low concentrations (in a similar range as in our study) did not reveal protective antioxidative effects (for example [18,23,24]). However, it should be stressed that the lack of in vitro effects does not exclude some positive effects in living organisms.

The question arises if the ovaries have higher estrogen activity and, consequently, stronger antioxidative properties, because estrogens can immediately affect the structures by which they are produced, and because the ovary has probably more estrogen receptors than the thyroid. Whereas this issue requires to be experimentally proven, our results do not support such a hypothesis.

It is worth mentioning that postmenopausal 17β-estradiol reduction is associated with the increased oxidative stress and the increased iron level [25], with the latter contributing potentially to the additional oxidative damage. It has been recently shown that postmenopausal women display significantly higher oxidative stress compared to women of reproductive age [26].

Other clinical conditions confirming protective effects of 17β-estradiol against damaging effects of iron relate to hemosiderosis or hemochromatosis, typically associated with iron overload. Hemochromatosis, a genetically-determined disease associated with excessive iron accumulation, is more prevalent in men than in women, and women are diagnosed around ten years later than men, mostly after menopause [20,27]. Additionally, in male patients with hemochromatosis, iron is accumulated in a larger quantity than in women with this entity [28]. This sexual dimorphism may be mainly caused by higher 17β-estradiol levels in premenopausal women than in men.

It is worth mentioning that in women with hemochromatosis (caused by, for example, a mutated *HFE* gene), thyroid diseases can be accompanied by pathological process in ovaries; C282Y mutation in the *HFE* gene may cause the development of epithelial ovarian cancer [29].

Another clinical condition associated with iron overload is folic acid deficiency. Treatment with folic acid of patients with iron overload was expectedly followed by decrease in serum and hepatic cell iron levels [30].

Considering the results of our work we can speculate that 17β-estradiol can reveal potential therapeutic effects in the treatment of iron overload. It should be remembered, however, that 17β-estradiol has prooxidative activity. It is known that high levels of 17β-estradiol are associated with a higher risk of estrogen-dependent cancer, such as breast and endometrial cancer [31,32]. In agreement with that, exogenous estrogens can increase oxidative damage in different tissues under experimental conditions [15,33], and even induce polycystic ovary syndrome [34].

Therefore, the use of 17β-estradiol, as a potential therapeutic agent, should be considered only in certain clinical conditions. However, treatment with 17β-estradiol in patients with lower than normal estrogen concentrations, e.g., in patients with premature ovarian failure, seems to be of great importance in the context of our present observations.

The finding that 17β-estradiol was protective to similar extent in the ovary (the place of physiological production of estrogens), as it was protective in the thyroid, strongly suggests that exogenous estrogens may reveal protective antioxidative effects in the whole organism.

This study has certainly some limitations. The first one is that our study is not an in vivo study and even we did not use the whole organs; instead, we have used tissue homogenates, so our results may not be directly extrapolated into in vivo conditions, especially in human populations. The other one is that the concentrations of 17β-estradiol, which revealed protective effects in our model, can be achieved only under experimental conditions.

## 5. Conclusions

Summarizing, the basal level of oxidative damage to membrane lipids is lower in the ovary than in the thyroid, however, with higher sensitivity of the former tissue to exogenous Fenton reaction substrates. Protective effects of 17β-estradiol against experimentally-induced lipid peroxidation, observed independently of iron concentration and tissue type, suggest that exogenous 17β-estradiol can be protective in various organs with iron overload. Potential therapeutic effect of 17β-estradiol against iron overload consequences should be considered.

## References

[1] Halliwell B. (1994). Free radicals and antioxidants: A personal view. Nutr. Rev..

[2] Koppenol W.H., Hider R.H. (2019). Iron and redox cycling. Do’s and don’ts. Free Radic. Biol. Med..

[3] Fenton H.J.H. (1894). LXXIII. Oxidation of tartaric acid in presence of iron. J. Chem. Soc. Trans..

[4] Lipinski B. (2011). Hydroxyl radical and its scavengers in health and disease. Oxid. Med. Cell. Longev..

[5] Sugawara M., Sugawara Y., Wen K., Giulivi C. (2002). Generation of oxygen free radicals in thyroid cells and inhibition of thyroid peroxidase. Exp. Biol. Med..

[6] Chen M.J., Chou C.H., Shun C.T., Mao T.L., Wen W.F., Chen C.D., Chen S.U., Yang Y.S., Ho H.N. (2017). Iron suppresses ovarian granulosa cell proliferation and arrests cell cycle through regulating p38 mitogen-activated protein kinase/p53/p21 pathway. Biol. Reprod..

[7] Karbownik M., Lewiński A. (2003). Melatonin reduces Fenton reaction-induced lipid peroxidation in porcine thyroid tissue. J. Cell. Biochem..

[8] Karbownik-Lewińska M., Kokoszko-Bilska A. (2012). Oxidative damage to macromolecules in the thyroid–Experimental evidence. Thyroid Res..

[9] Karbownik-Lewińska M., Stępniak J., Krawczyk J., Zasada K., Szosland J., Gesing A., Lewiński A. (2010). External hydrogen peroxide is not indispensable for experimental induction of lipid peroxidation via Fenton reaction in porcine ovary homogenates. Neuro Endocrinol. Lett..

[10] Stępniak J., Karbownik-Lewińska M. (2016). 17β-estradiol prevents experimentally-induced oxidative damage to membrane lipids and nuclear DNA in porcine ovary. Syst. Biol. Reprod. Med..

[11] Stępniak J., Lewiński A., Karbownik-Lewińska M. (2013). Membrane lipids and nuclear DNA are differently susceptive to Fenton reaction substrates in porcine thyroid. Toxicol. In Vitro..

[12] Stepniak J., Lewinski A., Karbownik-Lewinska M. (2019). Oxidative damage to membrane lipids in the thyroid -no differences between sexes. Drug Chem. Toxicol..

[13] Behl C., Skutella T., Lezoualc’h F., Post A., Widmann M., Newton C.J., Holsboer F. (1997). Neuroprotection against Oxidative Stress by Estrogens: Structure-Activity Relationship. Mol. Pharmacol..

[14] Prokai L., Prokai-Tatrai K., Perjesi P., Zharikova A.D., Perez E.J., Liu R., Simpkins J.W. (2003). Quinol-based cyclic antioxidant mechanism in estrogen neuroprotection. Proc. Natl. Acad. Sci. USA.

[15] Stepniak J., Lewinski A., Karbownik-Lewinska M. (2018). Sexual dimorphism of NADPH oxidase/H₂O₂ system in rat thyroid cells; effect of exogenous 17β-estradiol. Int. J. Mol Sci..

[16] Dahlman-Wright K., Cavailles V., Fuqua S.A., Jordan V.C., Katzenellenbogen J.A., Korach K.S., Maggi A., Muramatsu M., Parker M.G., Gustafsson J.A. (2006). International Union of Pharmacology. LXIV. Estrogen Receptors. Pharmacol. Rev..

[17] Santin A.P., Furlanetto T.W. (2011). Role of Estrogen in Thyroid Function and Growth Regulation. J. Thyroid Res..

[18] Culmsee C., Vedder H., Ravati A., Junker V., Otto D., Ahlemeyer B., Krieg J.-C., Krieglstein J. (1999). Neuroprotection by estrogens in a mouse model of focal cerebral ischemia and in cultured neurons: Evidence for a receptor-independent antioxidative mechanism. J. Cereb. Blood Flow Metab..

[19] Bradford M.M. (1976). A rapid and sensitive method for the quantitation of microgram quantities of protein utilizing the principle of protein-dye binding. Anal. Biochem..

[20] Bacon B.R., Adams P.C., Kowdley K.V., Powell L.W., Tavill A.S. (2011). Diagnosis and management of hemochromatosis: 2011 Practice Guideline by the American Association for the Study of Liver Diseases. Hepatology.

[21] Bresgen N., Eckl P.M. (2015). Oxidative stress and the homeodynamics of iron metabolism. Biomolecules.

[22] Abbassi-Ghanavati M., Greer L.G., Cunningham F.G. (2009). Pregnancy and laboratory studies: A reference table for clinicians. Obstet. Gynecol..

[23] Moosmann B., Behl C. (1999). The antioxidant neuroprotective effects of estrogens and phenolic compounds are independent from their estrogenic properties. Proc. Natl. Acad. Sci. USA.

[24] Vedder H., Anthes N., Stumm G., Würz C., Behl C., Krieg J.C. (1999). Estrogen hormones reduce lipid peroxidation in cells and tissues of the central nervous system. J. Neurochem..

[25] Jian J., Pelle E., Huang X. (2009). Iron and menopause: Does increased iron affect the health of postmenopausal women?. Antioxidants Redox Signal..

[26] Montoya-Estrada A., Velázquez-Yescas K.G., Veruete-Bedolla D.B., Ruiz-Herrera J.D., Villarreal-Barranca A., Romo-Yañez J., Ortiz-Luna G.F., Arellano-Eguiluz A., Solis-Paredes M., Flores-Pliego A. (2020). Parameters of oxidative stress in reproductive and postmenopausal Mexican women. Int. J. Environ. Res. Public Health.

[27] Barton J.C., Edwards C.Q. *HFE* Hemochromatosis. Synonyms: Hemochromatosis Type 1, *HFE*-Associated Hemochromatosis, HFE-HH. https://www.ncbi.nlm.nih.gov/books/NBK1440/.

[28] Edwards C.Q., Kelly T.M., Ellwein G., Kushner J.P. (1983). Thyroid disease in hemochromatosis. Increased incidence in homozygous men. Arch. Intern. Med..

[29] Gannon P.O., Medelci S., Le Page C., Beaulieu M., Provencher D.M., Mes-Masson A.M., Santos M.M. (2011). Impact of hemochromatosis gene (*HFE*) mutations on epithelial ovarian cancer risk and prognosis. Int. J. Cancer.

[30] Greenberg M.S., Grace N.D. (1970). Folic acid deficiency and iron overload. Arch. Intern. Med..

[31] Brinton L.A., Trabert B., Anderson G.L., Falk R.T., Felix A.S., Fuhrman B.J., Gass M.L., Kuller L.H., Pfeiffer R.M., Rohan T.E. (2016). Serum estrogens and estrogen metabolites and endometrial cancer risk among postmenopausal women. Cancer Epidemiol. Biomarkers Prev..

[32] Wang R., Li J., Yin C., Zhao D., Zhao Y., Li Y., Yin L. (2018). Role of β-estradiol in MCF-7 breast cancer cell line based on the bioinformatics analysis. Gynecol. Obstet. Investig..

[33] Karbownik M., Reiter R.J., Burkhardt S., Gitto E., Tan D.X., Lewiński A. (2001). Melatonin attenuates estradiol-induced oxidative damage to DNA: Relevance for cancer prevention. Exp. Biol. Med..

[34] Tang Z.R., Zhang R., Lian Z.X., Deng S.L., Yu K. (2019). Estrogen-receptor expression and function in female reproductive disease. Cells.

